# G2NPAN: GAN-guided nuance perceptual attention network for multimodal medical fusion image quality assessment

**DOI:** 10.3389/fnins.2024.1415679

**Published:** 2024-05-13

**Authors:** Chuangeng Tian, Lei Zhang

**Affiliations:** School of Information Engineering (School of Big Data), Xuzhou University of Technology, Xuzhou, China

**Keywords:** generative adversarial networks, image quality assessment, multimodal medical fusion image, perceptual, objective evaluation metrics

## Abstract

Multimodal medical fusion images (MMFI) are formed by fusing medical images of two or more modalities with the aim of displaying as much valuable information as possible in a single image. However, due to the different strategies of various fusion algorithms, the quality of the generated fused images is uneven. Thus, an effective blind image quality assessment (BIQA) method is urgently required. The challenge of MMFI quality assessment is to enable the network to perceive the nuances between fused images of different qualities, and the key point for the success of BIQA is the availability of valid reference information. To this end, this work proposes a generative adversarial network (GAN) -guided nuance perceptual attention network (G2NPAN) to implement BIQA for MMFI. Specifically, we achieve the blind evaluation style via the design of a GAN and develop a Unique Feature Warehouse module to learn the effective features of fused images from the pixel level. The redesigned loss function guides the network to perceive the image quality. In the end, the class activation mapping supervised quality assessment network is employed to obtain the MMFI quality score. Extensive experiments and validation have been conducted in a database of medical fusion images, and the proposed method is superior to the state-of-the-art BIQA method.

## 1 Introduction

**FIGURE 1:**
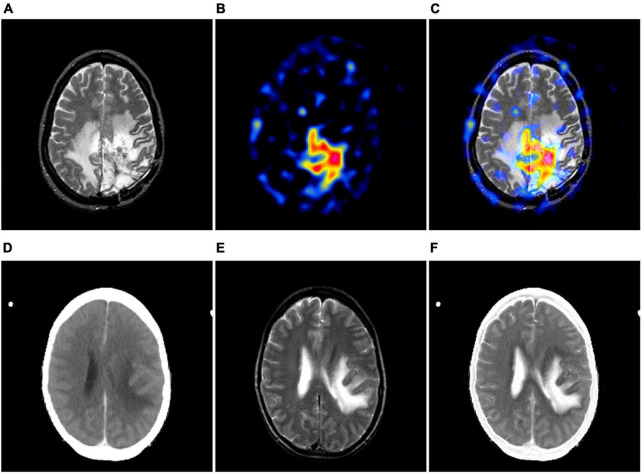
The examples of multimodal medical images. **(A, B)** are MR-T2-weighted image and the PET image of the same case; **(D, E)** are CT and MR-T2-weighted image of the same case. **(C, F)** show its corresponding image fusion results, respectively.

Over the past decade, medical images such as computed tomography (CT), magnetic resonance imaging (MRI), positron emission tomography (PET) and single photon emission computed tomography (SPECT) have played an increasingly important role in diagnosis, treatment, follow-up recommendations and intraoperative navigation of diseases (Zhou et al., 2020; He et al., 2023; Honkamaa et al., 2023). Depending on the theory of medical imaging techniques and the image features characterized by each modality, multimodal medical images can be simply divided into structural and functional images. The former can precisely locate the lesion and show the structural changes of the lesion, while the latter can sensitively reflect the physiological, biochemical and functional changes of the tissues and organs in the body, making it easier to detect the lesion. For instance, Figure 1A shows an MR image of a brain with glioma, from which the localization information and the internal structure of the tumor can be known, while the edematous region can be found to occupy almost more than half of the area of this tomography. Unfortunately, radiologists are not yet able to recognize the pathological features of the tumor from this image alone. Figure 1B shows the PET image of this case. The images of this modality do not have detailed information on brain structure, but it is very easy to identify the lesions with significant abnormal foci of radioactive concentration in the area of the lesion. Based on the imaging features of the above two modalities, the radiologists can then diagnose this disease and even complete a preliminary pathological grading, as shown in Figure 1C. Similarly, Figure 1 also displays a group of cerebral infarction cases where the images of the two modalities express different imaging features. As can be seen from the example, the diagnosis of a particular disease may require reference to multiple modalities at the same time. In view of the fact that the mono-modal image may not be enough to support the conclusion of disease diagnosis, some studies have gradually proposed to integrate the feature advantages of various modalities of medical images through image fusion technology. In addition, many literatures have reported that radiologists can significantly improve the accuracy of disease diagnosis, when they view medical images in multiple modalities simultaneously (Li and Zhu, 2020; Xu and Ma, 2021; Zhou et al., 2023).

Multi-modality medical image fusion (MMIF) is a technique that integrates the medical images obtained from two or more medical imaging devices, extracts the useful information from their respective modalities to maximize, and ultimately forms a comprehensive image (Zhang et al., 2020; Zhang G. et al., 2023; Liu et al., 2024b). Nowadays, image fusion methods specifically for the field of medical images have been vigorously developed, and various excellent fusion algorithms have also been proven in practice. However, due to the different principles of these fusion algorithms, the quality of the generated fusion images is uneven, which needs to be measured by a unified set of standards. Generally, the most direct way to assess the fused image is to have the fused image observed and analyzed by a radiologist. Although this subjective evaluation method can give a score consistent with the human visual system (HVS), but, the quality score of the fused image is influenced by the environment, and cannot be directly analyzed quantitatively due to the direct human involvement. More importantly, subjective assessment is a time-consuming and labor-intensive process (Lei et al., 2022; Liu et al., 2022). This would not be permissible in an already rushed clinical setting.

In contrast to the subjective evaluation, objective evaluation methods detect some indicators of the image to measure the quality of the fused image, such as mutual information (MI), peak signal-to-noise ratio (PSNR), or structural similarity (SSIM). These metrics have achieved excellent achievements in the field of natural image quality assessment (Wang J. et al., 2021). However, it is undeniable that these metrics tend to assess more general properties of images, and are not suitable for assessing medical fused images. This is primarily in a clinical setting, a medical fusion image with excellent quality may not be because it has a high signal-to-noise or anything, but because this fusion image effectively helps the physician to make a diagnostic decision. As mentioned earlier, each modality of medical images expresses unique imaging features. The traditional image quality evaluation methods may ignore the unique feature representation of medical fused images, resulting in the evaluation results that are inconsistent with those of radiologists. Such analytical findings motivated us to find way to represent such unique features when developing MMIF-specific quality evaluation metrics. Particularly, the mean opinion score (MOS) given by radiologists serves perfectly as the ground truth for the quality of fused images. If the network could learn the difference between images with lower and higher MOS, this will be more valuable for the model to assess the quality of the fused images. Furthermore, in practical application scenarios, a completely distortion-free fused reference image (i.e., optimal quality) is difficult, or even impossible, to obtain.

To overcome these problems, in this paper, we propose a GAN-guided nuance perceptual attention network (G2NPAN) for implementing blind image quality assessment (BIQA). A method specifically designed for quality assessment of multimodal medical fusion images.

Specifically, to learn the nuances between different fused images, we use a generative adversarial network (GAN). It has been realized in our previous work that effective spatial feature extraction techniques for image texture and shape can effectively improve the effectiveness of image quality assessment. Therefore, we designed an overlapping structure in the generator, named Unique Feature Warehouse (UFW), to learn spatial features of the fused images from the pixel level and enhance the ability of the network to learn the perception of quality differences between different fused images. Because the purpose of this paper is to accurately assess multimodal medical fusion images, rather than to obtain a perfect fusion image, we redesigned the loss function of the discriminator according to the clinical requirements for image quality. Although GAN can provide powerful guidance for the quality assessment of multimodal medical fusion images, it is still challenging to fully utilize such information. Therefore, we designed the attention-based quality assessment network (AQA) using the supervision of class activation mapping (CAM). Enabling AQA to utilize the fused images generated by GAN at higher resolution and also to sufficiently learn high-dimensional features at lower resolution.

To summarize, the main contribution of this work is in the following folds: (1) We propose a GAN-guided quality difference perceptual attention network. It can achieve accurate quality assessment of multimodal medical fusion images in a blind form. (2) In the generator, we developed the UFW module for learning fused image spatial features from the pixel level. (3) A loss function specifically for multimodal medical fusion image quality perception is designed based on a generative adversarial network architecture. (4) With the supervision of CAM, the proposed AQA is able to learn nonlinear mappings between fused images and objective quality results from lower and higher resolution, respectively, which further enhances the efficacy of model assessment.

The rest of this paper is organized as follows. Section 2 “Related work” introduces the related work of this paper. In Section 3 “Methodology”, the details of the proposed methods are described. The adequate experimental results and discussion are presented in Section 4. Finally, we summarize our conclusions at the end of the paper.

## 2 Related work

### 2.1 Multimodal medical image fusion (MMIF)

In the medical setting, mono-modality imaging cannot provide comprehensive body tissue information or lesion characteristics and is insufficient to support disease diagnosis (Wang et al., 2020). Therefore, multimodal medical image fusion technology has been created to improve the utilization of medical imaging information. This technology can be classified into traditional fusion methods and deep learning based fusion methods. Traditional image fusion techniques often face challenges with distortion, whereas deep learning-based methods for image fusion have seen notable advancements in recent years (Zhang, 2021; Wang A. et al., 2022; Karim et al., 2023). For example, Wang et al. (Wang Z. et al., 2022) designed a self-supervised residual feature learning network for multi-focus image fusion. Xu and Ma (2021) developed an unsupervised image fusion network with enhanced information preservation by surface-level and deep- level constraints. It is worth specifying that the network is built specifically for medical images. But, the performance of fusion algorithms and the quality assessment of fused images are not yet fully understood. Whether the multimodal image fusion technique can be successfully applied, the quality assessment of the fused images is the key.

### 2.2 Image quality assessment for MMIF

Based on the different requirements for reference images, the objective image quality assessment methods (IQA) can be divided into three categories: full-reference IQA (FR-IQA), reduced-reference IQA (RR-IQA), and no-reference IQA (NR-IQA) i.e., BIQA. Despite FR-IQA and RR-IQA methods have achieved remarkable success in the past decades, their application fields are restricted due to their dependence on reference images. This is because reference images are not always available in practical application scenarios, and even more, in some fields, it is almost impossible to obtain them. Therefore, BIQA has gained the favor of many researchers as it does not require any reference image for evaluation.

According to the way of feature extraction, BIQA includes: statistical analysis-based models and learning-based models. Most existing models based on statistical analysis attempt to detect concrete types of distortion, such as various forms of blur and noise. And the learning-based BIQA model aims to reflect the differences in image quality through effective feature extraction techniques as well as to design the model to learn the mapping relationship between features and image quality. Traditional machine learning approaches assume that either distortion will cause the image to change in some feature attributes. Therefore, this kind of method pays more attention to the process of feature extraction. The quality regression models are then designed by machine learning methods such as support vector machine (SVM), K-Nearest Neighbors algorithms, etc. Some classical models are, for example, BRISQUE (Mittal et al., 2012), NFERM (Gu et al., 2015) and BIBE (Wang et al., 2016).

However, those method separates the process of feature extraction and quality score prediction/regression of images. This leads to models that cannot be implemented in an end-to-end learning manner. Moreover, feature extraction schemes based on hand design rely on the experience of the researcher, and the features obtained from limited understanding of the image may not sufficient to describe the image content. Most recently, the vigorous development of deep learning techniques is gradually becoming the mainstream of IQA algorithms (Hou et al., 2015; Madhusudana et al., 2022; Zhang Z. et al., 2023; Liu et al., 2024a). Earlier, Kang et al. (2014) integrated feature learning and regression into an optimization process by a simple CNN architecture and obtained promising generalization results. In (Wang X. et al., 2021), WANG et al. proposed a novel tone-mapped image metric using local degradation characteristics and global statistical properties. Inspired by the observer subjective assessment process, Sim et al. (2022) proposed a novel BIQA algorithm based on the semantic recognition task. Yue et al. (2023) implemented an automated assessment of colonoscopy images by analyzing brightness, contrast, colorimetry, naturalness, and noise. But BIQA methods specifically for multimodal medical fusion images have not been adequately explored. Considering the absence of referenceable fusion images in a real clinical setting, we design a novel learning-based BIQA model.

### 2.3 GAN-based image quality assessment

In the process of image quality assessment, since the reference image is not always available, it poses a great difficulty in constructing a learning-based IQA model (Liu et al., 2019). Until 2014, the emergence of GAN has brought new ideas to researchers in many fields. GAN could attempt to generate better outputs with adversarial training of generators and discriminators. Therefore, if the reference image can be generated for the BIQA method, it will be possible to bridge the performance gap between the FR-IQA and BIQA methods. Moreover, the concern that standard reference images for multimodal fusion images are not available in the clinical setting will be mitigated. A series of GAN-based work has also been carried out by researchers related to image quality evaluation (Ma et al., 2019; Guo et al., 2023; Kelkar et al., 2023; Li and He, 2024). In 2019, Ma et al. (2019) proposed an end-to-end GAN model for quality assessment of images based on multitasking. And the superiority of the method was verified in TID2008 and TID2013 datasets. The same year, Yang et al. (2019) designed a BIQA method with the advantages of self-generated samples and self-feedback training, called BIQA-GAN. GAN-based methods have the ability to learn local distortion characteristics and whole quality on the depth features of the image, and it can accomplish the mapping fitting of potential features to the target domain. Thus, we introduce GAN to design our model, and, we tuned the loss function and architecture of GAN according to the characteristics of medical images.

## 3 Methodology

In this section, we introduce an end-to-end no-reference method, namely GAN-guided nuance perceptual attention network (G2NPAN), for assessing the quality of multimodal medical fusion images. First, we introduce the framework of the proposed method. Then, we elaborate on the two main parts of our proposed G2NPAN, i.e., the GAN-guided nuance perceptual module and attention-based quality assessment network. Finally, the quality perception loss function is formulated.

### 3.1 Overview

**FIGURE 2:**
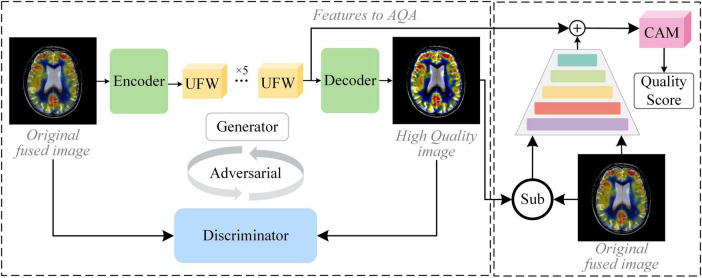
The framework of the proposed GAN-guided nuance perceptual attention network.

The core idea of our proposed method is to assume that high MOS fused images can indeed help the physician in clinical analysis. Therefore, learning the nuance between lower and higher MOS fused images is of high value for quality assessment in the absence of a reference image. The framework of G2NPAN is shown in Figure 2. Briefly, our method is specified below. Firstly, G2NPAN learns the nuances between the fused images with different quality through generative adversarial networks, and utilizes generators to generate fused images with the best possible quality. For the generator, we carefully designed an overlapping structure, UFW, and repeated it five times to increase the ability to learn the nuances between different fused images. Then, we redesigned the loss function of the discriminator according to the scoring criteria of images in the clinical setting. The aim is to increase the perceptual weight of the image quality during the network training process. Next, we subtract the high MOS image generated by the generator from the original fused image to obtain the difference between them. Finally, we feed this nuance together with the original fused image into an attention-based quality assessment network to obtain a nonlinear mapping between the fused image and the objective quality results. We will describe this process in detail in the remaining part of this section.

### 3.2 GAN-guided nuance perceptual module

#### 3.2.1 GAN architecture

GAN is a distinctive approach to achieving feature extraction by generating new fused images in the form of generative adversarial. This network structure normally consists of two main parts, the Generator (**G**) and the Discriminator (**D**). On the one hand, GAN has domain adaptive property. For non-discrete distribution data, like fused images, it is more robust for feature extraction or learning. On the other hand, the generator can generate fused images of the same type through adversarial training, and under the supervision of the discriminator, the generated images are fitted toward higher quality. The proposed G2NPAN is established on the framework of GAN, which takes the original fused image as input and passes the image nuance information to obtain a quality score of fused images. It is worth noting that the purpose of high-quality fused images produced by the generator is to provide reference information, which may contribute to the quality assessment of the original fusion image. More specifically, it may help to alleviate the problems associated with the absence of reference images.

**FIGURE 3:**
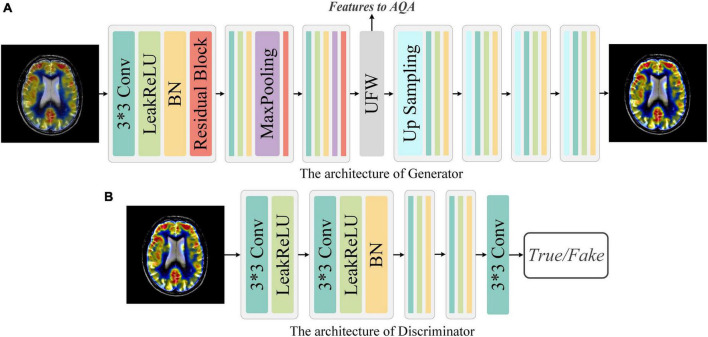
Network architecture of Generator and Discriminator. **(A)** Generator: The Generator is a simple down-sampling and up-sampling convolution neural network with the Unique Feature Warehouse. **(B)** Discriminator: The Discriminator consists of a simple five-layer convolution neural network.

Our network structure of the generator and discriminator is presented in Figure 3. **G** takes the fused image *I*_*org*_ with arbitrary quality as input and aims to generate a fused image *I*_*hq*_ with the best quality, i.e., *I*_*hq*_ = *G*(*I*_*org*_). The discriminator exists to distinguish the real fused image *I*_*org*_ from the generated version of the fused image *I*_*hq*_. Through adversarial training, it is expected that the fused image with the best quality can be generated with the arbitrary quality of fused image as input.

**Generator:** As shown in Figure 3, **G** is a convolution neural network consisting of down-sampling and up-sampling phases. Since medical fused images require the network to focus on more detailed features, the kernel size in the generator is all set to 3 × 3. The down-sampling operation is composed of three sequential networks with the same structure. Specifically, we connect the convolutional layer, the activation function, the batch normalization (BN), and the residual block to build this sequential network. For the activation function, a comprehensive activation algorithm, Lleaky Rectified Line Unit (LeakyReLU), is used. To make the model more stable, the BN layer is attached after the activation layer, which can also help the gradient to back propagate efficiently. For medical fused images, the conventional simply increasing the depth of the convolution neural network may cause the model to converge slowly or even be unable to converge. Therefore, we invoke the residual structure in the down-sampling process, which consists of three convolution layers and a skip connection. After the down-sampling operation, followed by our elaborate UFW structure, which improves the ability of the network to learn the nuances between different fused images. We will describe the detailed structure of UFW in the next subsection. In the up-sampling phase, we designed a simple four-layer convolutional neural network. Each layer of the convolutional neural network consists of an up-sampling operation, a convolutional with a kernel size of 3 × 3, a batch normalization, and an activation layer. As for the activation function, we use the LeakReLU activation function in the first three layers and the Sigmoid activation function in the last layer. So far, the best quality image *I*_*hq*_ of size 128 × 128 can be obtained by using the *I*_*org*_ as the input. The generator’s parameters are only renewed by the mean squared error (MSE) and are defined as in Eq. 1:


(1) $$L_{1}=\frac{1}{N}\,\sum_{n=1}^{N}{\left(G\,\left(I_{o\,r\,g}\right)-I_{G\,T}\right)}^{2}=\frac{1}{N}\,\sum_{n=1}^{N}{\left(I_{h\,q}-I_{G\,T}\right)}^{2},$$

where *N* is the total number of generated samples. *I*_*GT*_ means the fused image with high MOS, i.e., Ground Truth (GT).

During the training of **G**, the following objective function (Eq. 2) is minimized:


(2) $$L_{G}={𝔼}_{I_{o\,r\,g}∼P_{d\,a\,t\,a\,O}}\,\left[\log\left(1-D\,\left(I_{G\,T},G\,\left(I_{o\,r\,g}\right)\right)\right)\right]+\theta \,L_{1},$$

where P_*dataO*_ stands for the data distribution of *I*_*org*_, and the 𝔼_*Iorg*_∼*P*_*dataO*_ represents the expectation of *I*_*org*_. θ is a weighted hyperparameter.

**Discriminator:** The discriminator only needs to judge whether the image conforms to the real data distribution or not. Thus, the architecture of discriminator is a simple four-layer convolution neural network, as illustrated in Figure 3. In brief, each network has one convolutional layer with a kernel size of 3 × 3, a stride of 2, and padding of 1. Then LeakyReLU is used as the activation function and subsequently processed with BN. Note that with each layer of the convolutional neural network, the size of the feature map shrinks to one-fourth of the input. Finally, we add an independent convolution layer according to the sequential structure, which is mainly used for classification. The mean absolute error is used as the loss function to optimize the parameters of discriminator. Thus, the objective function of discriminator can be expressed as Eq. 3:


(3) $$L_{D}={𝔼}_{I_{G\,T}∼P_{d\,a\,t\,a\,G\,T}}\left[\log D\left(I_{G\,T}\right)\right]+{𝔼}_{I_{o\,r\,g}∼I_{P_{d\,a\,t\,a\,O}}}[\log\left(1-D\left(G\left(I_{o\,r\,g}\right)\right)\right],$$

where P_*dataGT*_ is the data distribution of *I*_*GT*_, and 𝔼_*IGT*_∼*P*_*dataGT*_ is the expectation of *I*_*GT*_.

#### 3.2.2 Unique feature warehouse (UFW)

**FIGURE 4:**
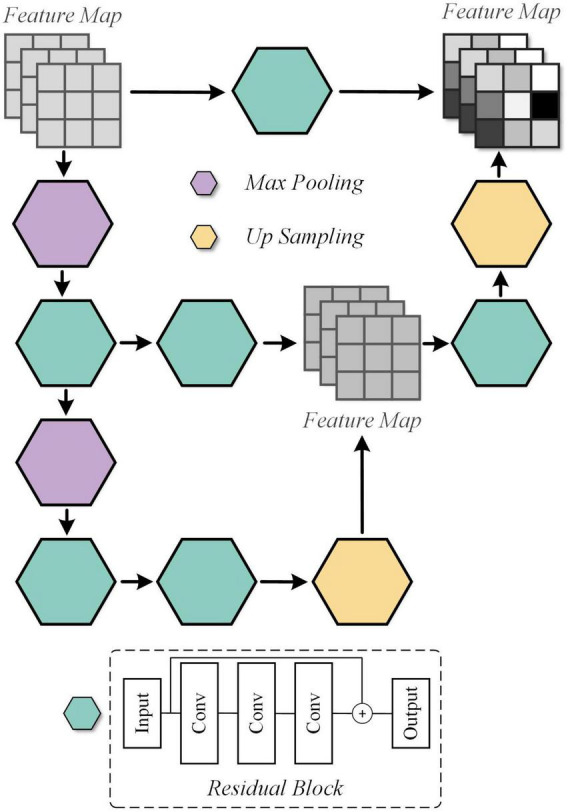
Detailed architecture of the Unique Feature Warehouse (UFW) module.

In our previous work, it has been realized that effective spatial feature extraction techniques for image texture and shape play an important role in the quality assessment of medical fusion images. The preservation of anatomical details, the representation of metabolic information, and the trade-offs of information during the fusion process are one of the many characteristics to be recognized in fused image assessment. Thus, an overlapping structure, UFW, is designed in this paper to enable the model to capture these features from fused images at multiple scales. The detailed architecture of the UFW is presented in Figure 4. Note that both the input and output feature maps are 32 × 32. On the one hand, taking a full-resolution image as input requires a large amount of memory consumption. On the other hand, most of the high-dimensional features appear only at lower resolutions. Therefore, we embedded the UFW module at the end of the down-sampling stage of the generator, with a maximum resolution of 32 × 32. In addition, with the overlapping architecture, the network can process the high-dimensional features multiple times to further learn and weigh their relationship. Consistent with the design purpose of the generator, the kernel size we use in UFW are all 3 × 3 to better focus on subtle spatial features. The UFW structure recognizes image spatial features from multiple scales and continuously integrates them in the overlapping structure to achieve effective spatial feature extraction.

### 3.3 Attention-based quality assessment network

**FIGURE 5:**
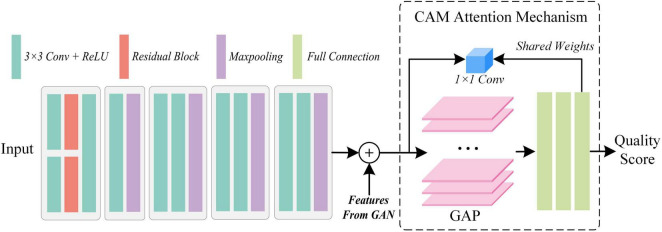
The architecture of the proposed attention-based quality assessment network.

The attention-based quality assessment network is built on the VGG network, which is a simple convolutional neural network as shown in Figure 5. The reason for adopting VGG network are respectively: the VGG network is an easy-to-use CNN, which can save a lot of effort in modifying its network architecture. Also, with the guidance of GAN, AQA is not required to extract the feature representation of the fused image from scratch. Therefore, it is less necessary to employ a complex network structure. Finally, VGG11, which has a relatively simple structure and shallow network depth in VGG networks, was used as the base framework in the AQA. AQA takes the nuance between the original fused image and the generated image, and the original fused image as input to obtain an objective assessment of the fused image.

Specifically, from the structure of GAN this paper takes the fused image with higher MOS as GT of the generator, thus limiting its fitting trend. Thus, the nuance between the fused image *I*_*org*_ and the higher quality fused image *I*_*hq*_ can be defined as *I*_sub_ = |*I*_*hq*_ − *I*_*org*_|. *i*denoting the *i-th* assessed image, the definition can be revised to Eq. 4:


(4) $$I_{s\,u\,b}^{i}=\left|I_{h\,q}^{i}-I_{o\,r\,g}^{i}\right|,$$

To ensure the input consistency, $I_{s\,u\,b}^{i}$and $I_{o\,r\,g}^{i}$performed the convolution operation first separately, and then completed the concatenation operation.

For $I_{o\,r\,g}^{i}$, its features extracted in the GAN can also guide the AQA to obtain more convincing assessment results. So, we copied the feature map **F**_*last*_ output from the last UFW module, and implemented concatenation before inputting the fully connected layer to obtain the feature map *F*_*conca*_, as shown in Eq. 5:


(5) $${\text{F}}_{c\,o\,n\,c\,a}={\text{F}}_{l\,a\,s\,t}⊙v\,g\,g\,\left(I_{o\,r\,g}^{i},I_{s\,u\,b}^{i}\right),$$

where *vgg*(●) denotes the operation of AQA before proceeding to the fully connected layer.

So far, the image quality assessment has been achieved objectively by the methods mentioned above. However, for physicians, the quality of medical images depends *not only* on the natural nature of the images, *but also* on their ability to highlight the manifestations of disease. The latter is the key to assist doctors in making a diagnosis. Thus, by using the weights of the last fully connected layer as a cue, we introduce the attention mechanism, class activation mapping. With the quality scores of AQA and the weights of the fully connected layers, CAM obtains the ability to supervise the attention distribution of the network. Moreover, the feature map **F**_*CAM*_ generated by CAM can also compensate for the un-interpretability of “black box” models. Let the GT of CAM be **F**_*GT*_, then the objective function is Eq. 6:


(6) $$L_{C\,A\,M}=\frac{1}{N}\,\sum_{n=1}^{N}{\left|{\text{F}}_{C\,A\,M}-{\text{F}}_{G\,T}\right|}_{1},$$

where |●|_1_ denotes the L1 parametrization.

Further, the predicted score of the fused image is designated as *Q*_*pre*_ and its GT is *Q*_*t*_, then the objective function can be written as shown in Eq. 7:


(7) $$L_{Q\,A}=-\left[Q_{t}\,\log\left(\sigma \,\left(Q_{p\,r\,e}\right)\right)+\left(1-Q_{t}\right)\,\log\left(1-\sigma \,\left(Q_{p\,r\,e}\right)\right)\right],$$

where σ(●) denotes the sigmoid function, which is meant to map *Q*_*pre*_ to the interval (0, 1), specified as shown in Eq. 8:


(8) $$\sigma \,\left(Q_{p\,r\,e}\right)=\frac{1}{1+e\,x\,p\,\left(-Q_{p\,r\,e}\right)},$$

Thus, the loss function of AQA can be expressed as Eq. 9:


(9) $$L_{A\,Q\,A}=\varphi \,L_{C\,A\,M}+L_{Q\,A},$$

φ is the weight parameter.

### 3.4 Perceptual loss function

The design logic of GAN is trained in an adversarial way so that the generated image can deceive the discriminator, and the discriminator can distinguish the real image from the generated image. Although such a network architecture can generate high-quality fused images, the ultimate goal of G2NPAN is to accurately evaluate the quality of fused images rather than to obtain fused images. Moreover, it is clear from the calculation of $I_{s\,u\,b}^{n}$ that it depends heavily on the generated image *I*_*hq*_ . If $I_{s\,u\,b}^{n}$ is directly used as the input of AQA without feedback to GAN, the training process of quality assessment network will be unstable and difficult to converge. Thus, we design the quality perception loss function to alleviate the above problem. It is worth clarifying that the fused images used in this work are based on a further extension of the database from our previous work (Tang et al., 2020), and thus the MOS of each medical fused image can take values from 1 to 5. Typically, ensuring that the MOS remains above 3 does not compromise the diagnostic results provided by medical professionals. This ensures that the fused medical images do not adversely impact diagnostic performance. Therefore, the weight can be expressed as Eq. 10:


(10) $$W^{n}=\left\{ \begin{array}{cc} 1, & \text{if }\,A\,Q\,A\,\left(I_{h\,q}^{\text{n}}\right)\geq 3,\\ 0, & \text{if }\,A\,Q\,A\,\left(I_{h\,q}^{n}\right)<3 \end{array}\right.,$$

We the weight to further optimize the network and restate the formula (3) as shown in Eq. 11 below:


(11) $$L_{D}={𝔼}_{I_{G\,T}∼P_{d\,a\,t\,a\,G\,T}}\,\left[\log D\,\left(I_{G\,T}\right)\right]+{𝔼}_{I_{o\,r\,g}∼I_{P_{d\,a\,t\,a\,O}}}$$

$$\left[\log\left(1\;-\text{ |}D\left(G\left(I_{org}\right)\right)\;-\;W|\right)\right],$$

The generated images need to be distinguished not only by the discriminator, but also by the quality assessment network. The concept of perceptual loss function allows the model to be optimized as a unit, so that the total loss function can be presented as Eq. 12:


(12) $$L_{a\,l\,l}=\min_{G}\max_{D}V\,\left(G,D\right)+\gamma \,L_{R},$$

## 4 Experiments

### 4.1 Databases and experimental protocols

#### 4.1.1 Dataset description

We established a multi-modal medical image fusion quality evaluation database to validate the effectiveness of our proposed algorithm. The database comprises 120 pairs of color images and 9 pairs of grayscale images, with a total of 1,290 images generated using 10 mainstream fusion algorithms. The resolution of the images is 128 × 128 pixels. The MOS of each image was obtained from radiologists on a scale ranging from 1 to 5.

To select reference images for each group of fused images (i.e., 10 images generated by fusing a pair of images), we used MOS to evaluate image quality. The fused image with the highest MOS score was chosen as the reference image. If multiple fused images had the highest MOS score, one of them was selected at random. The reference image for each image was randomly selected from the fused images with the highest MOS score. This ensured a robust reference image selection process that accounted for the subjective quality ratings of the radiologists.

### 4.1.2 Evaluation criteria

In this study, we utilized four evaluation metrics to assess the performance of the proposed model: Pearson’s Linear Correlation Coefficient (PLCC), Spearman’s Rank Correlation Coefficient (SRCC), Kendall’s Rank Correlation Coefficient (KRCC), and Root Mean Square Error (RMSE). PLCC measures the linear relationship between the predicted and the corresponding MOS, while SRCC and KRCC are non-parametric correlation measures that evaluate rank-based data. RMSE measures the difference between the predicted and the corresponding MOS.

#### 4.1.3 Experimental protocols

In the training process of the network, three hyperparameters, θ, φ, and γ, were set to 0.5, 1, and 0.01, respectively. The Adam optimizer was used with an initial learning rate of 0.0002. Furthermore, we implemented a dynamic learning rate adjustment strategy to enhance model convergence during training. Specifically, we reduced the learning rate using a decay factor of 0.95 after every 20 batches.

To evaluate the effectiveness of our proposed model, we employed a five-fold cross-validation approach during implementation. We use 80% images of the database to train our model, while using 20% to test. The model’s performance was evaluated at the end of each training epoch, and we selected the checkpoint model with the best performance within the 1500 epochs of training as the final model. During the validation phase, we assessed the model’s performance on the test set. In each evaluation, we try 1000 times and take an average of the performance values obtained.

### 4.2 Comparison with the state-of-the-art

In this section, exhaustive comparative experiments are conducted to validate our proposed method. We compared the performance of G2NPAN with the performance of six state-of-the-art BIQA methods. For approaches that are not specifically named, we refer to them by the name of the first author. All these methods include the blind multiple pseudo reference images-based method (BMPRI) (Min et al., 2018), In-depth analysis of Tsallis entropy-based method (TEIA) (Sholehkerdar et al., 2019), mutual information-based optimization method (Hossny) (Hossny et al., 2008), the objective evaluation of fusion performance (OEFP) (Xydeas and Petrovic, 2000), ratio of spatial frequency error-based method (rSFe)(Zheng et al., 2007) and the perceptual quality assessment method (Tang) (Tang et al., 2020). BMPRI introduces multiple pseudo-reference images to achieve BIQA, which coincides with our approach of using GAN to generate reference information to perform IQA. Thus, although BMPRI is not specifically developed for quality assessment of fused images, it is still used as one of the comparison methods. And the remaining methods are proposed exclusively for the quality assessment of fused images. Note that the proposer of rSFe considers the application scenario of medical fusion images, while the Tang method is proposed especially for medical fusion images. For fair comparison, all methods were retrained and tested in our Dataset, and the best results were used as the final reported.

We have tabulated the performance of the state-of-the-art BIQA method and G2NPAN in Table 1. The best performance results are highlighted in bold. Based on Table 1, we have the following observations:

**TABLE 1:** Performance comparison with Other BIQA methods.

Model	Domain	PLCC	SRCC	KRCC	RMSE
BMPRI	Distorted image	0.3031	0.3167	0.2375	0.2611
TEIA	Fused image	0.1797	0.1946	0.1407	0.3909
Hossny	Fused image	0.2270	0.1738	0.1071	0.3712
OEFP	Fused image	0.3064	0.3367	0.2342	0.2810
rSFe	Fused image	0.4054	0.2275	0.1700	0.2663
Tang	Fused image	0.6252	0.6420	0.4166	0.2480
**Proposed**	Fused image	**0.9044**	**0.9007**	**0.8502**	**0.1029**

Bold values represent the best results.

First, our proposed method, G2NPAN, achieved the best quality assessment performance from an overall perspective, with optimal results of 0.9044, 0.9007, 0.8502, and 0.1029 for PLCC, SRCC, KRCC and RMSE, respectively. This means that the objective evaluation results derived from the G2NPAN are closest to the subjective MOS results given by the physicians. Second, although the BMPRI method introduces pseudo-reference images to provide referenceable information for BIQA, it is mainly targeted at distorted images of natural scenes. Therefore, it is not powerful for medical fusion images. Our proposed method generates reference information based on high-quality fused images and designs quality evaluation methods from the specificity of medical images, resulting in the best BIQA performance. As can be seen from Table 1, BMPRI also outperforms some of the quality evaluation metrics designed specifically for fused images, which once again demonstrates the importance of reference information for BIQA. Third, rSFe, Tang and the proposed method have considered the difference between medical fusion images and natural fusion images, and thus their performance is better than the other three metrics (TEIA, Hossny and OEFP). In addition, the performance of proposed method is still 27.92, 25.87, 43.36, and 14.51% better than the second-best method in PLCC, SRCC, KRCC and RMSE, respectively. From the above analysis, it is clear that our proposed G2NPAN method is very good at objective quality assessment of medical fusion images.

### 4.3 Ablation study

Ablation experiments are performed from different perspectives to demonstrate the superiority of our proposed method. (1) To verify the generalizability of the proposed AQA, we compose the model through different backbone networks, including VGG11, VGG16, VGG19, and tested the model performance. Each ablation result is demonstrated in Table 2, with the best result for the corresponding metric highlighted in bold. (2) To evaluate the contribution of each key component in the proposed G2NPAN model, a series of ablation experiments were conducted.

**TABLE 2:** Ablation experiments of quality assessment with different backbone networks.

Network	PLCC	SRCC	KRCC	RMSE
VGG19	0.7530	0.7358	0.6696	0.1601
VGG16	0.7776	0.7742	0.6977	0.1553
VGG11	0.7894	0.7905	0.7132	0.1494
VGG11 + CAM	0.8000	0.7806	0.7173	0.1385
VGG 11 + pre	0.8167	0.8112	**0.7519**	0.1355
VGG11 + pre + CAM	**0.8235**	**0.8113**	0.7496	**0.1330**

Bold values represent the best results.

#### 4.3.1 Performance of quality assessment network

Ablation studies were performed to examine whether the backbone network used in the quality prediction network was more appropriate. All models implemented in this section are purely quality prediction networks, meaning that there is no GAN-based quality guidance. Their testing performance is listed in Table 2.

On the one hand, from these results, we can notice that the performance of the VGG11 is even better than that of VGG16 or VGG19. This seems to go against the common belief that the deeper the network, the better the model performance should be. But there should be more detailed analysis for different task types. The truth is that VGG19 or 16 has more convolutional layers than VGG11, which allows the network to learn more semantic information (high-level features). However, for evaluation of multimodal medical fusion images, the model does not need to recognize what the image represents, like what disease or which organ, etc., but rather than what the image has. Thus, the IQA task might require more structural (low-level features) than semantic information about the image. VGG11 improved by 3.64, 5.47, 4.36, and 1.07% in PLCC, SRCC, KRCC and RMSE, respectively, compared to VGG19.

On the other hand, to demonstrate the usefulness of the CAM and pre-trained models, we have adapted them based on the VGG11 model. From the experimental results, it can be seen that both CAM and the introduced pre-trained model enhance the performance of quality prediction network. And, based on these two techniques, the proposed quality prediction network achieves 0.8235, 0.8113, 0.7496, and 0.1330 in PLCC, SRCC, KRCC and RMSE, respectively.

#### 4.3.2 The contribution of each key component

As mentioned in the previous section, the proposed method integrates the nuances between fused images with different qualities, and adjusts the update of the loss function according to the scoring criteria of images in the clinical setting. Therefore, it is sensible and meaningful to fully explore the contribution of each key component to the final performance.

**FIGURE 6:**
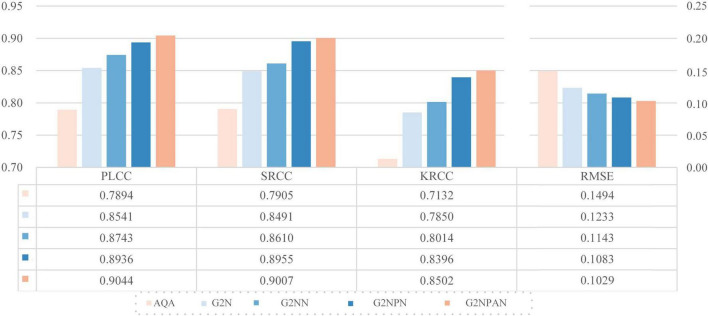
Ablation experimental results for each of the key components in G2NPAN.

We take VGG11 network as the base quality prediction model. Based on this, the GAN-guided quality assessment models with and without UFW components are named G2N (GAN-Guided Network) and G2NN (GAN-Guided Nuance Network), respectively. Further, we redesigned the loss function of GAN in a qualitatively perceptive way. Such a modified network named G2NPN (GAN-Guided Nuance Perceptual Network). Eventually, CAM is added to the G2NPN model to supervise the quality prediction results of the fused images and call such a network G2NPAN (GAN-Guided Nuance Perceptual Attention Network), i.e. the model proposed in this paper. Note that we trained G2N, G2NN, and G2NPN based on the same method applied in G2NPAN and summarized their corresponding prediction performance results in Figure 6. As all three models, G2N, G2NN and G2NPN, are degradation models based on GAN tuning, the blue family is used for unification in Figure 6.

As expected, all key components had a positive effect on the final model performance. And as the model structure becomes closer to G2NPAN, the quality assessment of the medical fusion images becomes more accurate. Further analysis is as follows. First of all, with the reference information provided by GAN, the G2N model achieves the largest performance improvement over VGG11 with 6.47, 5.86, 7.18, and 2.61% improvement in PLCC, SRCC, KRCC and RMSE, respectively. The G2N model generates the best-quality fused image similar to providing the reference image for IQA, and thus, it has the most significant performance improvement. However, the nuances in the reference information might not be sufficient. UFW is an effective way to extract spatial features by learning the features of fused images from multiple scales several times. Therefore, the G2NN model further enhances the performance results. Second, as the GAN has the ability to recognize the quality of fused images, i.e., the perceptual capability, the G2NPN model obtains considerable performance gains, especially in SRCC (0.8610 *vs* 0.8955) and KRCC (0.8014 *vs* 0.8396). Finally, by introducing the CAM attention mechanism, our proposed G2NPAN has got the best performance for medical fusion image quality assessment, with PLCC, SRCC, KRCC and RMSE of 0.9044, 0.9007, 0.8502, and 0.1029, respectively.

Overall, whether it is the visual impression of the blue rectangular bar in Figure 6 or the data analysis results, it can be found our proposed GAN-guided approach could yield a tremendous performance improvement. Except for RMSE, the improvement results for the other three metrics were more than 10%. It is also interesting to observe that the models with reference information provided by GAN outperform all the methods shown in Table 2.

### 4.4 Impact of training set

To investigate the relationship between the sample size and the performance of the proposed method, we gradually increased the training sample size from 20 to 80%, while the rest of the image samples were used as testing. All experimental results are filled in Table 3. It is intuitive to notice that as the training sample size increases, the proposed model performance tends to rise gradually. And, the model performance does not drop precipitously when the training sample size are smaller. This observation is consistent with the conclusions drawn from the existing learning-based BIQA (Gu et al., 2016; Jiang et al., 2019; Wang X. et al., 2021). The robustness of the proposed G2NPAN model has been validated.

**TABLE 3:** Performance on the dataset with various train-test splits.

Train: Test	PLCC	SRCC	KRCC	RMSE
2:8	0.7780	0.7849	0.7042	0.1649
3:7	0.8055	0.8148	0.7372	0.1441
4:6	0.8497	0.8451	0.7817	0.1275
5:5	0.8621	0.8617	0.8044	0.1206
6:4	0.8821	0.8795	0.8259	0.1139
7:3	0.8848	0.8886	0.8393	0.1093
8:2	**0.9044**	**0.9007**	**0.8502**	**0.1029**

Bold values represent the best results.

## 5 Conclusion

In this paper, we propose a BIQA method specifically for multimodal medical fused images, called GAN-Guided Nuance Perceptual Attention Network. Specifically, in addition to designing the UFW module in the GAN to incorporate collecting useful features from the pixel level, we also redesigned the loss function of the discriminator to enable the network to learn the nuance between fused images of variable quality. Following that, the nuance information and the high-dimensional features in the UFW are fed back to the quality assessment network. With the supervision of CAM, the quality score of the fused image is eventually determined. The experimental results demonstrated that our proposed method outperforms the state-of-the-art methods. Two aspects of ablation experiments validate the generality of the proposed AQA and the contribution of each key component of the G2NPAN model. The experiments examining the correlation between sample size and G2NPAN performance further verify the effectiveness of the proposed GAN-guided quality assessment model.

## Data availability statement

The original contributions presented in this study are included in this article/supplementary material, further inquiries can be directed to the corresponding author.

## Author contributions

CT: Methodology, Writing−original draft. LZ: Validation, Writing−review and editing.
